# Long-Term Exposure to Ambient Air Pollution and Mortality Due to Cardiovascular Disease and Cerebrovascular Disease in Shenyang, China

**DOI:** 10.1371/journal.pone.0020827

**Published:** 2011-06-10

**Authors:** Pengfei Zhang, Guanghui Dong, Baijun Sun, Liwen Zhang, Xi Chen, Nannan Ma, Fei Yu, Huimin Guo, Hui Huang, Yungling Leo Lee, Naijun Tang, Jie Chen

**Affiliations:** 1 Department of Biostatistics, School of Public Health, China Medical University, Shenyang, People's Republic of China; 2 Department of Occupational and Environmental Health, School of Public Health, China Medical University, Shenyang, People's Republic of China; 3 Shenyang Center for Diseases Control and Prevention, Shenyang, People's Republic of China; 4 Department of Occupational Health, College of Public Health, Tianjin Medical University, Tianjin, People's Republic of China; 5 Institute of Epidemiology and Preventive Medicine, College of Public Health, National Taiwan University, Taipei, Taiwan; University of Tor Vergata, Italy

## Abstract

**Background:**

The relationship between ambient air pollution exposure and mortality of cardiovascular and cerebrovascular diseases in human is controversial, and there is little information about how exposures to ambient air pollution contribution to the mortality of cardiovascular and cerebrovascular diseases among Chinese. The aim of the present study was to examine whether exposure to ambient-air pollution increases the risk for cardiovascular and cerebrovascular disease.

**Methodology/Principal Findings:**

We conducted a retrospective cohort study among humans to examine the association between compound-air pollutants [particulate matter <10 µm in aerodynamic diameter (PM_10_), sulfur dioxide (SO_2_) and nitrogen dioxide (NO_2_)] and mortality in Shenyang, China, using 12 years of data (1998–2009). Also, stratified analysis by sex, age, education, and income was conducted for cardiovascular and cerebrovascular mortality. The results showed that an increase of 10 µg/m^3^ in a year average concentration of PM_10_ corresponds to 55% increase in the risk of a death cardiovascular disease (hazard ratio [HR], 1.55; 95% confidence interval [CI], 1.51 to 1.60) and 49% increase in cerebrovascular disease (HR, 1.49; 95% CI, 1.45 to 1.53), respectively. The corresponding figures of adjusted HR (95%CI) for a 10 µg/m^3^ increase in NO_2_ was 2.46 (2.31 to 2.63) for cardiovascular mortality and 2.44 (2.27 to 2.62) for cerebrovascular mortality, respectively. The effects of air pollution were more evident in female that in male, and nonsmokers and residents with BMI<18.5 were more vulnerable to outdoor air pollution.

**Conclusion/Significance:**

Long-term exposure to ambient air pollution is associated with the death of cardiovascular and cerebrovascular diseases among Chinese populations.

## Introduction

Ambient air pollution significantly increases both morbidity and mortality in the general population [1]–[6]. The development of respiratory diseases resulting from direct contact of the respiratory system with air pollutants has been widely acknowledged as a major component of the adverse health effects of air pollution [7]. However, during the past two decades, air pollution-induced cardiovascular and cerebrovascular toxicities have become the focus of intensive studies among cardiologists and specialists in environmental medicine [8]–[11]. Despite these studies, the relationship between ambient air pollution and cardiovascular and cerebrovascular diseases is still controversial. This is especially true for cerebrovascular diseases, since findings in available studies have been inconsistent [10]–[13].

The contribution of exposure to air pollution to human cardiovascular and cerebrovascular diseases seems to vary in different parts of the world, because of the differences in spatial and temporal variability of air pollutant sources and components between the different regions [14]–[15]. For example, during 1980s, the major sources of ambient particulate pollutants in Shenyang, a city in northern China, were industrial emissions and the burning of smoky coal burning in residential areas; however, in Beijing, the sources were domestic smokeless coal burning and natural dust [16]. The results of chemical analyses of domestic coal components suggest that smoky coal contains much higher concentrations of toxic materials compared with smokeless coal [17]. In recent years, it has been reported that lignites from a local Shenyang coal field had the highest concentrations of nickel (75 µg/g) and chromium (79 µg/g) in the world, and some carcinogenic substances in coal were released into the air during the combustion of lignites in Shenyang [18]. It has also been reported that various factors, such as sex, age, education, and occupational exposure, can modify the relationship between air pollution and mortality [19], and the effects of air pollution exposure on health are greater in people with lower socioeconomic status (SES) [20]. Moreover, most of these studies were conducted in developed countries, and only a small number of studies have been conducted in Asia. Therefore, there is a need for studies conducted in developing countries, where the characteristics of ambient air pollution (e.g., air pollution level and mixture and transport of pollutants), meteorological conditions, and sociodemographic patterns may differ from those in North America and Europe.

Shenyang, the largest and a heavily industrialized city in China, was notorious for its heavy air pollution during the 1980s and 1990s. It had been reported that during an 11–year period (1981–1991), the annual, ambient total suspended particulate (TSP) level in Shenyang was 461 µg/m^3^. Thoracic particles (those less than 10 µm in diameter [PM_10_]) contributed to 70% of total TSP levels [21]. These values were approximately 5- to 10-fold greater than the concentrations of TSP or PM_10_ reported in US cities [22]. However, since the late 1990s, ambient air quality in China has improved as a result of environmental protection requirements. For example, about 1000 chimneys, located in residential communities, used for domestic heating in winter have been dismantled each year since 2001; as of 2006, a total of about 6000 chimneys have been knocked down. In addition, thousands of industries that were located throughout the city and contributed to ambient air pollution have been closed down or moved out of Shenyang during the past 10 years. Despite the significant improvements in ambient air quality in Shenyang, air pollution continues to pose a challenge to regulatory and health professionals. The concentrations of traditional pollutants (PM_10_ and sulfur dioxide [SO_2_]) remain higher than those in major cities in the United States, Western Europe, some Asian countries, such as Singapore and Japan, and other cities in China, including Hong Kong. Shenyang is also developing quickly; the numbers of automobiles have increased from 300,000 in 2000 to 800,000 in 2009, worsening the air pollution from traffic. Consequently, air pollution in Shenyang has been compounded, overlapping soot air pollution with traffic air pollution. This situation provides a unique opportunity to examine exposures to ambient air pollution in relation to the population's cardiovascular and cerebrovascular health in Shenyang city, China.

We conducted a population-based, retrospective cohort study to estimate the long-term effects of air pollutants (PM_10_, SO_2_, and nitrogen dioxide [NO_2_]) on mortality from cardiovascular and cerebrovascular diseases in Northern China, with a specific interest in factors that may modify the effect, including sex, age, education, and SES. Results of this study will provide a scientific basis for making public policies, controlling air pollution, setting air quality standard, and risk assessment.

## Results

### Description of the study subjects

Table 1 shows the detailed demographic characteristics of the subjects included in this study. A total of 12 584 residents were enrolled in the study, and 9941 subjects (79%) completed the medical questionnaire. The mean age of enrolled subjects was 58.09 years (range, 35–103 years), and 51.47% were female. Almost of subjects were of the Han nation and were married. A low educational level was achieved by 71.48% of the participants, and 40.63% of these reported that they were smokers or former smokers. The average BMI was 22.36 kg/m^2^ (SD, 3.49).

**Table 1:** Characteristics of the study population in the five districts of Shenyang city, 1998–2009.

Characteristic	Deaths			Subjects (%)
	Cardiovascular (%)	Cerebrovascular (%)	Total (%)	
Samples	138	118	256	9941
Age				
< = 60	21(15.22)*	10(8.47)*	31(12.11)*	5880(59.15)
>60	117(84.78)	108(91.53)	225(87.89)	4061(40.85)
Gender				
Male	102(73.91)*	85(72.07)*	187(73.05)*	4824(48.53)
Female	36(26.09)	33(27.93)	69(26.95)	5117(51.47)
Nation				
Han	135(97.83)	113(95.76)	248(96.88)	9449(95.05)
Other	3(2.17)	5(4.24)	8(3.13)	492(4.95)
Marital status				
Married	127(92.03)	105(88.98)	232(90.63)	8921(89.74)
Unmarried‡ †	11(7.97)	13(11.02)	24(9.38)	1020(10.26)
Educational level				
Low	94(68.12)*	89(75.42)*	183(71.48)*	5970(60.05)
High	44(31.88)	29(24.58)	72(28.52)	3971(39.95)
Personal income(RMB)#				
<200/month	22(15.94)*	26(22.03)	48(18.75)	1817(18.35)
(200, 500)/month	34(24.64)	37(31.36)	71(27.73)	3081(31.11)
(500, 800)/month	29(21.01)	33(27.97)	62(24.22)	2386(24.10)
> = 800/month	53(38.41)	22(18.64)	75(29.30)	2618(26.44)
Smoking habits				
No smoking	67(48.55)*	47(39.83)*	114(44.53)*	4359(43.85)
Second-hand smoking	25(18.12)	13(11.42)	38(14.84)	2732(27.48)
Smoking	46(33.33)	58(49.15)	104(40.63)	2850(28.67)
Occupational exposure				
Yes	7(5.07)	9(7.63)	16(6.25)	820(8.25)
No	131(94.93)	109(92.37)	240(93.75)	9121(91.75)
BMI(kg/m^2^)▵				
BMI<18.5	13(9.42)	8(6.78)*	21(8.20)*	1010(10.16)
18.5≤BMI<25	90(65.22)	72(61.02)	162(63.28)	6981(70.22)
25≤BMI	35(25.36)	38(32.20)	73(28.52)	1950(19.62)
Exercises				
Yes	69(50.00)*	56(47.46)	125(48.83)*	4146(41.71)
No	69(50.00)	62(52.54)	131(51.17)	5795(58.29)

*Significantly different from respective control.

# 39 subjects were not reported their personal income.

† Include separated, divorced, single, and widowed.

‡ Low, illiterate, primary and junior high school; high, high school or above.

▵ BMI, Body mass index.

There were 138 death due to cardiovascular diseases and 118 deaths from cerebrovascular diseases. The mortality rates from cardiovascular diseases and cerebrovascular diseases were 1.39% (95% CI, 1.16–1.62) and 1.19% (95% CI, 0.98–1.40), respectively, among subjects 38 to 100 years old at study entry; 50% of the deaths were among those who were 69 to 79 years old.

### Characteristics of air pollution

Table 2 shows the mean annual levels of PM_10_, SO_2_, and NO_2_ in each of the representative districts where air pollution was measured. During the study period, the mean annual level of PM_10_ was 154 µg/m^3^ (SD, 41 µg/m^3^) and ranged from 78 to 274 µg/m^3^. For SO_2_, the mean level was 63 µg/m^3^ (SD 15 µg/m^3^), with a range of 26 to 106 µg/m^3^. And for NO_2_, the mean level was 46 µg/m^3^ (SD 13 µg/m^3^), with a range of 18 to 78 µg/m^3^.

**Table 2:** Air pollution levels in the five districts of Shenyang city in 1998–2009 (µg/m^3^).

Years	Dadong District			Tiexi District			Heping District			Shenhe District			Huanggu District		
	PM_10_	SO_2_	NO_2_	PM_10_	SO_2_	NO_2_	PM_10_	SO_2_	NO_2_	PM_10_	SO_2_	NO_2_	PM_10_	SO_2_	NO_2_
1998	214	41	56	274	106	74	239	92	75	236	70	72	155	46	47
1999	207	46	58	237	97	71	225	78	78	204	83	67	122	49	40
2000	188	53	52	188	66	42	192	67	49	190	67	44	116	41	42
2001	183	51	51	199	73	53	189	75	60	189	72	57	132	50	40
2002	167	52	48	170	45	46	166	72	50	166	56	50	146	48	34
2003	159	53	45	135	48	35	163	71	53	171	73	53	108	42	25
2004	155	58	44	164	55	40	140	68	49	155	73	51	138	57	37
2005	146	60	42	139	50	36	136	66	46	153	74	49	124	60	37
2006	135	63	45	136	50	33	113	73	48	140	81	56	125	64	43
2007	137	65	33	120	51	34	131	56	38	151	76	42	116	72	39
2008	119	67	35	93	32	23	97	59	35	126	76	43	119	68	36
2009	110	70	33	78	26	18	83	57	31	117	76	41	116	70	35

Overall, PM_10_, SO_2_, and NO_2_ levels were relatively highly correlated with each other. The Pearson's correlation coefficient for mean annual PM_10_ and SO_2_ levels was 0.49 (P<0.01); for PM_10_ and NO_2_ levels, 0.88 (P<0.01); and for SO_2_ and NO_2_ levels, 0.65 (P<0.01).

Annual mean PM_10_ levels decreased during the study period in all areas, but was most dramatic in the Tiexi district, where the, PM_10_ decline was an average of 16 µg/m^3^ per year. For SO_2_ and NO_2_, most, but not all annual mean levels decreased during the same period in every district, with changes in the Tiexi district the most pronounced.

### Factors associated with mortality

Based on univariate analysis, a significant association was seen between mortality from both cardiovascular disease and cerebrovascular disease and age, sex, educational level, smoking habits, BMI, and exercise frequency. There were no statistically significant differences between mortality and nation, marital status, personal income, and occupational exposure. A separate analysis of the two diseases failed to find a significant association between the mortality from either cardiovascular or cerebrovascular diseases and nation, marital status, or occupational exposure. However, there was a statistically significant association between mortality from cardiovascular disease and personal income, but not for BMI. For cerebrovascular disease, no significant association was seen for exercise frequency. (Table 1)

Table 3 shows the results of the Cox regression analysis for the impact of air pollution exposure on both cardiovascular and cerebrovascular disease mortality, adjusted for confounders. A multivariate analysis was conducted for each pollutant using the mean levels for the period from 1998 to 2009. After adjusting for age, sex, BMI, educational level, smoking habits, personal income, occupational exposure, and exercise frequency, PM_10_ and NO_2_ were significantly associated with mortality from the two diseases, but there no significant association between cardiovascular and SO_2_. After excluding air pollutants from the model, significant associations were observed between mortality from cardiovascular disease and age, sex, personal income, occupational exposure, and exercise frequency. For cerebrovascular diseases, associations with age, sex, personal income, smoking habits, BMI, and exercise frequency were statistically significant. The HRs with 95% CIs are described in Table 3.

**Table 3:** Factors associated with mortality of cardiovascular and cerebrovascular from Cox proportional hazards model.

Factors	Cardiovascular		Cerebrovascular		All deaths	
	HR	95%C.I.	HR	95%C.I.	HR	95%C.I.
Age	1.08	(1.07,1.09)	1.11	(1.11,1.12)	1.10	(1.09,1.10)
Gender	0.35	(0.30,0.40)	0.44	(0.38,0.52)	0.39	(0.35,0.43)
Educational level	0.87	(0.76,1.01)	0.92	(0.77,1.09)	0.90	(0.80,1.00)
Personal income	1.21	(1.13,1.29)	0.93	(0.86,0.99)	1.07	(1.02,1.12)
Smoking habits	0.94	(0.88,1.01)	1.41	(1.30,1.53)	1.13	(1.07,1.19)
Occupational exposure	0.57	(0.42,0.77)	1.13	(0.89,1.44)	0.82	(0.68,0.98)
BMI(kg/m^2^)▵	0.93	(0.83,1.04)	1.50	(1.32,1.69)	1.15	(1.06,1.25)
Exercises	0.64	(0.57,0.73)	0.54	(0.47,0.62)	0.59	(0.54,0.65)
PM_10_ *	1.55	(1.51,1.60)	1.49	(1.45,1.53)	1.53	(1.50, 1.56)
SO_2_ *	0.96	(0.92,1.01)	0.95	(0.90,1.00)	0.95	(0.92,0.99)
NO_2_ *	2.46	(2.31,2.63)	2.44	(2.27,2.62)	2.45	(2.34,2.58)

*HR (Hazard Ratios) associated with 10 µg/m^3^ changes in each pollutant, respectively, and was adjusted for age, gender, educational level, family, smoking status, personal income, occupational exposure, body mass index, and exercises.

▵ BMI, Body mass index.

The modifying effects of age, sex, educational level, smoking habits, personal income, occupational exposure, BMI, and exercise frequency on the association between both PM_10_ and NO_2_ and mortality were statistically significant, including SO_2_ and subjects with occupational exposure and little regular exercise. The HRs with 95% CI are described in Table 4.

**Table 4:** Estimated hazard ratios for cardiovascular and cerebrovascular associated with an increase of 10 µg/m^3^ in the level of PM_10_, SO_2_ and NO_2_.

Factors	PM_10_		SO_2_		NO_2_	
	HR*	95%C.I.	HR	95%C.I.	HR	95%C.I.
Age						
< = 60	1.58	(1.49,1.67)	0.96	(0.87,1.06)	2.51	(2.19,2.88)
>60	1.52	(1.49,1.55)	0.96	(0.93,1.00)	2.46	(2.34,2.59)
Gender						
male	1.50	(1.47,1.54)	0.95	(0.91,1.00)	2.37	(2.24,2.51)
female	1.59	(1.53,1.65)	0.96	(0.90,1.03)	2.68	(2.45,2.95)
Educational level						
Low	1.55	(1.51,1.58)	0.94	(0.90,0.98)	2.50	(2.36,2.65)
High	1.48	(1.43,1.53)	0.98	(0.92,1.06)	2.31	(2.12,2.53)
Smoking habits						
No smoking	1.58	(1.53,1.62)	0.94	(0.89,0.99)	2.56	(2.38,2.75)
Second-hand smoking	1.49	(1.42,1.56)	0.93	(0.85,1.02)	2.33	(2.05,2.65)
Smoking	1.49	(1.45,1.53)	0.98	(0.92,1.03)	2.38	(2.20,2.57)
Personal income(RMB)						
<200/month	1.47	(1.41,1.54)	0.90	(0.82,0.98)	2.42	(2.16,2.72)
(200, 500)/month	1.51	(1.45,1.56)	0.90	(0.84,0.96)	2.42	(2.20,2.66)
(500, 800)/month	1.64	(1.57,1.71)	1.03	(0.96,1.11)	2.64	(2.39,2.91)
> = 800/month	1.49	(1.44,1.55)	0.98	(0.91,1.05)	2.35	(2.16,2.57)
Occupational exposure						
Yes	1.71	(1.56,1.86)	1.22	(1.06,1.39)	3.22	(2.62,3.97)
No	1.52	(1.49,1.55)	0.94	(0.90,0.97)	2.42	(2.30,2.54)
BMI(kg/m^2^)▵						
BMI<18.5	1.69	(1.57,1.82)	0.84	(0.73,0.96)	2.41	(2.02,2.86)
18.5≤BMI<25	1.54	(1.50,1.57)	0.95	(0.91,0.99)	2.51	(2.36,2.67)
25≤BMI	1.47	(1.42,1.52)	1.00	(0.93,1.07)	2.34	(2.14,2.56)
Exercises						
Yes	1.47	(1.43,1.51)	0.82	(0.79,0.87)	2.13	(1.99,2.28)
No	1.59	(1.55,1.64)	1.11	(1.05,1.17)	2.83	(2.63,3.03)

*HR(Hazard Ratios) associated with 10 µg/m^3^ changes in each pollutant, respectively, and was adjusted for age, gender, educational level, family, smoking status, personal income, occupational exposure, body mass index, and exercises.

▵ BMI, Body mass index.

## Discussion

The results of the present study give strong support to the link between ambient air pollution exposure and the risk of mortality from both cardiovascular and cerebrovascular diseases. The main strength of this present study is that it is a retrospective cohort study and that it includes information about relevant confounders such as smoking, BMI, history of occupational exposure, and exercise frequency. Information on covariates and potential confounders was collected during face-to-face interviews, ensuring no self-reporting bias. Most other studies conducted in China were either conducted before the 1990s or were primarily concerned with the respiratory system and short-term effects. To our knowledge, this study represents the first time a Cox proportional hazards regression model was used to evaluate the effects of long-term air pollution exposure on cardiovascular and cerebrovascular mortality in China. In addition, the study subjects lived in one city and near air pollution measurement points, avoiding the possibility of between-city heterogeneity. Critics of earlier studies have suggested that poorly measured or unmeasured confounding factors may vary from city to city and account for, at least in part, the observed city-to-city differences in mortality rates associated with air pollution [16].

As reported in several studies conducted in other countries [2], [8], [12], [19], [23], [24], this present study found a positive association between PM_10_ and NO_2_ levels and cardiovascular and cerebrovascular mortality; however, the HRs in the present study were much higher than those seen in other studies. For example, in the United Kingdom, an increase in cerebrovascular mortality of 37% was observed between the lowest and highest quintile groups of modeled NO_2_ (means, 47.6 µg/m^3^ and 61.9 µg/m^3^, respectively) [23]. From a study conducted in Norway, Nafstad and colleagues reported an HR for cardiovascular deaths in men of 1.08 (95% CI, 1.03–1.12) for every 10 µg/m^3^ increase in NO_2_; the value of NO_2_ varied from 11.5 to 21.7 µg/m^3^. The HR for cerebrovascular deaths was 1.04 (95% CI, 0.94–1.15) [24]. However, in our study, the HRs for cardiovascular and cerebrovascular mortality were 2.46 (95% CI, 2.31–2.63) and 2.44 (95% CI, 2.27–2.62) respectively, associated with a 10 µg/m^3^ increase in NO_2_; the levels of NO_2_ in our study were similar to the levels reported in the study from the United Kingdom. Thus, based on results reported here, it is possible that any discrepancies in the effects of air pollutants could be related to differences in the pollutant composition between regions. In recent years, it has been reported that lignites from a local Shenyang coal field had the highest concentrations of nickel and chromium in the world, and some carcinogenic substances in coal were released into the air during the combustion of lignites in Shenyang [18].

The reasons for the lack of association between SO_2_ and any cerebrovascular or cardiovascular mortality in this present study are not clear. The effects of SO_2_ might have been masked by those of NO_2_ and PM_10_—the levels of both of these were fairly high in comparison. Kan and colleagues studied the short-term effects of air pollution on mortality in Shanghai during 2001 to 2004. The mean daily level of SO_2_ at nine monitoring stations was 44.7 µg/m^3^, a value below the level observed in our present study, and cardiovascular mortality was significantly associated with an increase in SO_2_ levels after adjustments for temperature, humidity, and seasons [19]. However, no significant association was seen with SO_2_ in studies conducted in St. Louis, Missouri, and eastern Tennessee in the United States, where the annual SO_2_ level at that time was similar to the levels found in our present study [25]. Also, some studies indicated that SO_2_ is more closely associated with respiratory mortality than PM_10_, whereas PM_10_ is associated more closely with cardiovascular mortality [12], [21]. Results from studies conducted in Hong Kong found that SO_2_, rather than PM_10_, was associated with deaths from pneumonia and influenza, especially when the mean level of SO_2_ was relatively low and that of PM_10_ was fairly high [12]. Therefore, susceptibility to pollutants can be affected by factors that influence dosimetry or by the response of tissues to ambient pollutant burdens. The mechanisms by which the various size and constituents of ambient pollutants could exert or modify health effects of cerebrovascular or cardiovascular diseases are not understood. Frampton and colleagues hypothesized that systemic inflammation and vasoconstriction with expression of leukocytes, endothelial adhesion molecules, oxidants, and interleukins can be induced by changes in vascular function due to particle acidity or particles with transition metals or ultrafine particles [26]. Also, it has been suggested that air pollution induces a low-grade pulmonary inflammatory response and subsequent release of pro-inflammatory cytokines. This may result in increased coagulability of the blood, triggering cardiovascular events in susceptible individuals [27]. For example, interleukin-6 is released from the bronchial mucosa and stimulates hepatic production of fibrinogen. There is also an association between respiratory symptoms and ischemic heart disease, further supporting the link between airway inflammation and cardiovascular disease [28].

We found a greater effect of ambient air pollution on cerebrovascular and cardiovascular mortality in women than in men. Results of prior studies on sex-specific effects of outdoor air pollution have been discordant. For example, Kan and colleagues [19] found the highest risk of mortality related to air pollution exposure among women. Hong et al [29] found that elderly women were most susceptible to the adverse effects of PM_10_ for the risk of acute mortality from stroke. However, Cakmak et al [30] found that sex did not modify the risk of hospitalization from cardiac diseases due to air pollution exposure. This finding is plausible, as there are differences between the male and female airways from early fetal lung development and throughout life [31], [32]. In addition, women have slightly greater airway reactivity than men, as well as smaller airways [33]; therefore, a dose-response relationship might be detected more easily in women than in men. Moreover, deposition of particles in the lung varies by sex, with a greater percentage of lung deposition of 1-µM particles in all lung regions for women [34], [35]. Sunyer et al. [36] has suggested that differing particulate deposition patterns between women and men may partly explain the difference between the sexes.

In contrast to the findings of the Women's Health Initiative (WHI) observational study, which showed that the association between cardiovascular events and the level of PM_2.5_ increased with increasing BMI and waist-to-hip ratio [37], we observed a stronger association between PM_10_ and SO_2_ and mortality of cerebrovascular and cardiovascular diseases among the individuals with a BMI less than 18.5 kg/m^2^ than for those with a normal BMI or a BMI of 25 kg/m^2^ or greater. The reasons for our BMI-specific observations are unclear and deserve further investigation. Our results also showed that the effect estimates of PM_10_ and NO_2_ on mortality in nonsmokers were slightly higher than in smokers. One study suggested that the effects of air pollution may be stronger in nonsmokers than in smokers [38]. Oxidative and inflammatory effects of smoking may dominate to such an extent that the additional exposure to air pollutants may not further enhance the effects along the same pathways in smokers.

The role of SES has received attention in air pollution epidemiology. In the present study, we did not find educational level or household income to modify the effects of air pollution on mortality from cerebrovascular and cardiovascular diseases. Similarly, results from the WHI observational study showed that neither educational level nor household income significantly affected the relationship between air pollution and cardiovascular disease, although there was a trend toward greater effects among those with less education [37]. Some studies have indicated that SES may modify the effects of air pollution, with much a stronger modification among the less educated [19], [39]. The cause of this interaction is not well understood. Most of the studies believed that persons with lower SES have a higher prevalence of preexisting diseases that confers a greater risk of mortality associated with air pollution exposure, and they may also receive inferior medical treatment for preexisting diseases. Moreover, they may not sufficient income to purchase foods known to be high in antioxidants, such as fish, fresh fruits, and vegetables, resulting in a reduced intake of antioxidants as well as polyunsaturated fatty acids and vitamins that may protect against the adverse consequences of particle exposure [40]. These individuals were more likely to live near busy roadways and have co-exposures due to either poor housing or occupation [41].

Finally, two main limitation of the present study should be noted. First, the design of the study, with recruitment taking place in 2009, may have led to bias because the events of interest may have already occurred. Secondly, we have been unable to control for weather factors (e.g., temperature or humidity) in this analysis. As noted by previous studies, many experts found a significant association between both temperature and humidity and cerebrovascular and cardiovascular mortality [42]. However, our analysis was unable to consider the impact of urban heat islands because of data inconsistencies between suburban and inner city weather stations. It is possible that this type of error may introduce bias to the results of our analysis. For example, in the ‘Sauna’ day in summer, individuals with poor economic conditions may lack air conditioning and lower heat foods, which could contribute to mortality. Therefore, future studies need to incorporate mesoscale meteorological models to overcome this limitation. In addition, compared with other studies in Europe and North America, our data were limited to only one city.

In conclusion, the present study confirmed that ambient air pollution was significantly associated with cerebrovascular and cardiovascular mortality in northeast China. These findings indicate that although significant improvements have been achieved in terms of air quality in the past decade in Shenyang, much more needs to be done to further reduce air pollution levels and associated diseases. Moreover, these findings have implications for regulatory and environmental policies, including implementation of measures to reduce ambient air pollution.

## Methods

### Ethics statement

The procedures followed were in accordance with ethical standards of the responsible committee on human experimentation of Tianjin Medical University. The ethics committee and other relevant regulatory bodies in Tianjin Medical University approved the study. A written informed consent was obtained from each participant before data collection.

### Sample design

During April 2009 to August 2009, a population-based, retrospective cohort study was conducted in Shenyang, in northeast China (longitude: 122°25′ to 123°48′; latitude: 41°12′ to 42°17′). The altitude of the residential area is 50 m above the mean sea level, and the mean annual air temperature is 6.5°C (range, −37.4°C to 38.0°C). Shenyang is comprised of both urban and suburban districts and counties, with a total area of 13 308 km^2^ and a population of 10 million as of 2007. The major industries include steel manufacturing, nonferrous metals, machinery, chemical- and coke-related industries, and electric power generation. Our study area was limited to the traditional five urban districts of Shenyang (230 km^2^). The target population included all permanent residents living in the area, approximately 5.1 million individuals in 2007.

There are five air monitoring sites located in five urban districts of Shenyang. In March of 2009, two communities within 1000 m of one of these monitoring sites were randomly selected from each district, and 500 to 700 families were then randomly selected from each of these communities. The entry criterion was that families needed to reside at the present location for at least 10 years (since January 1, 1998). Families who strictly met this criterion were included in the study. The participants were limited to members of the selected families who were at least 25 years of age in 1998 (or were born before January 1, 1973). The resulting 10 communities and a cohort of approximately 13 000 subjects were followed for 12 years, from 1998 through 2009. Trained interviewers administered a standardized questionnaire to all cohort members. They interviewed subjects directly, when feasible, or by surrogate respondents for subjects decreased or not present. The questionnaire elicited information on demographics (age, sex, and residential area), residential history, lifetime use of household stove and fuel types, SES (education level, marital status, and annual household income), cigarette smoking, alcohol consumption, diet, occupational history, exercise frequency, and medical history for 1998 and for 2009. Exercise was defined by reports of the subjects or families of the index subject's ever having taken some exercise outside (including: jogging, walking, shadowboxing, or ball sports) and/or inside (including swimming, aerobics, or ball sports), and has/had ≥2 exercises per week. Subjects were classified into three groups based on the smoking conditions: smoking, secondhand smoke, and nonsmoker. Smoking was defined by reports of the subjects of families of the index subject's ever smoking ≥1 cigarettes per day and for at least 6 months a year. Secondhand smoke has been defined as the smoke which non smokers are exposed to when they are in an indoor environment with smokers, and has/had ≥1 environmental tobacco smoke exposures per week. The questionnaire interview typically took about 30 minutes.

The cohort was followed until January 1, 2009. Deaths among the cohort during the study period were identified using death certifications, local public security bureaus, and local public health bureaus. In the event of a death in Shenyang, the decedent's family needed to obtain a death certificate from the hospital or local community clinic and submit it to the police station to cancel the decedent's household registration. In addition, the decedent's family needed to submit the death certificate to the local public health station, which in turn “sterilizes” the decedent's home. The decedent's family, therefore, obtains two certificates (i.e., one from the police station and the other from the local public health station), both of which were required for cremation. Information abstracted from the death records included name, sex, date of birth, residence, and date and cause of death. The name, sex, date of birth, and residence information was used to create a unique identifier for members of the cohort. Through this personal identifier and a link with the National Cause of Death Register, it was possible to identify subjects who had died from cardiovascular or cerebrovascular disease. The International Classification of Diseases, 9^th^ revision (ICD-9) categories 390 to 459 and 10^th^ revision (ICD-10) categories I00–I99 were used for the diagnosis of cardiovascular disease and categories 430–438 (ICD-9) and I60–I69 (ICD-10) were used for cerebrovascular disease.

### Air Pollution Data

**Figure 1:**
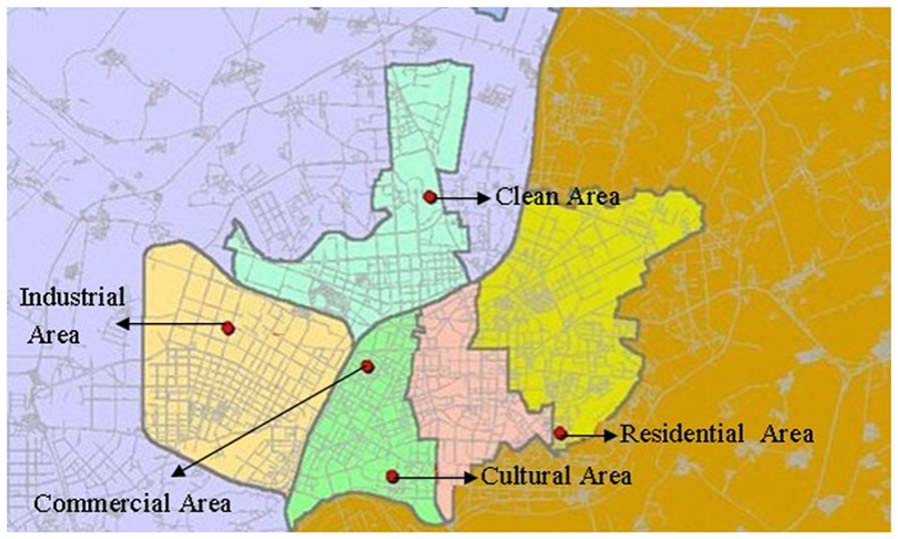
WHO's GEMS sites in the urban areas by the Shenyang Environmental Monitoring Center.

Beginning in 1981, TSPs and SO_2_ have been monitored in Shenyang under the direction of the World Health Organization (WHO)/United Nations Environment Program (UNEP) Global Environmental Monitoring System (GEMS). Since January 1, 1995, outdoor PM_10_, SO_2_, and NO_2_ levels were measured by the Shenyang Environmental Monitoring Center (the governmental agency in charge of collecting air pollution data in Shenyang) at GEMS sites in five representative urban areas: (1) Tiexi district (industrial), (2) Dadong district (residential), (3) Heping district (commercial), (4) Shenhe district (cultural), and (5) Huanggu district (clean) (see Figure 1). The daily levels for each pollutant were averaged from the available monitoring results of five fixed-site stations in the representative urban districts and covered by China National Quality Control. These stations are mandated to be located away from major roads, industrial sources, buildings, or residential sources of emissions from the burning of coal, waste, or oil; thus, our monitoring results reflect the background urban air pollution level in Shenyang rather than from local sources, such as traffic or industrial combustion. Levels of each pollutant were measured continuously and reported hourly—PM_10_ by beta-attenuation, SO_2_ by ultraviolet fluorescence, and NO_2_ by chemiluminescence. Exposure parameters in the present study were the 1-year average, and the yearly deviations in each station were calculated from the 24-hour NO_2_, SO_2_, and PM_10_.

### Statistical analysis

The Cox proportional-hazards regression was used to estimate hazard ratios (HR) and 95% confidence intervals (CIs) for the time to mortality from cardiovascular and cerebrovascular diseases associated with an increase of 10 µg/m^3^ in the level of long-term exposure to PM_10_, SO_2_, and NO_2_. In all models, factors hypothesized a priori that could potentially confound the relationship between air pollution and cardiovascular disease and cerebrovascular disease were included. These factors included age, body mass index (BMI), smoking status, the number of cigarettes smoked per day, the number of years of smoking, educational level, household income, and race or ethnic group. “Time-dependent covariate effect” was analyzed using a Cox regression model for counting processes. In this current study, subjects utilized at different time and different time points and with different pollutants exposure levels. This model can handle time-dependent covariates as well as left-truncation and right censoring while controlling for risk factors. All tests of significance were two-sided, and a 5% significance level was used throughout. Data management was carried out using Epidata 3.01 (Epidata Association), and statistical analyses were conducted using SAS software 9.13 (SAS Institute, Inc., Cary, NC, USA).
